# Evaluation of treatment efficacy of the ^161^Tb- and ^177^Lu-Labeled GRPR antagonists AMTG and RM2 in a mouse model of prostate cancer

**DOI:** 10.1007/s00259-026-08035-6

**Published:** 2026-07-10

**Authors:** Thomas Günther, Abraham S. Moses, Elisabeth Günther, Madeleine R. Landry, Stijn Ramaekers, Michiel Van de Voorde, Maarten Ooms, Bernard Ponsard, Corinne Beinat

**Affiliations:** 1 Molecular Imaging Program at Stanford (MIPS), Department of Radiology, School of Medicine, Stanford University, Stanford, CA USA; 2 University Duisburg-Essen and DKTK partner site University Hospital Essen, Department of Nuclear Medicine, Essen, Germany; 3 Nuclear Medical Applications, Belgian Nuclear Research Centre, Mol, Belgium; 4 BR2 Reactor, Belgian Nuclear Research Centre, Mol, Belgium

**Keywords:** AMTG, RM2, ^161^Tb (terbium-161), ^177^Lu (lutetium-177), GRPR

## Abstract

**Background:**

Recent data on ^161^Tb-labeled radiopharmaceuticals indicate a benefit in treatment efficacy due to the emission of high-energy Auger and conversion electrons in addition to β particles. We aimed to investigate differences in therapeutic potential of the gastrin-releasing peptide receptor (GRPR) antagonists RM2 (DOTA-Pip^5^-D-Phe^6^-Gln^7^-Trp^8^-Ala^9^-Val^10^-Gly^11^-His^12^-Sta^13^-Leu^14^-NH_2_) and AMTG (DOTA-Pip^5^-D-Phe^6^-Gln^7^-*α*-Me-Trp^8^-Ala^9^-Val^10^-Gly^11^-His^12^-Sta^13^-Leu^14^-NH_2_) radiolabeled with ^161^Tb and ^177^Lu in PC-3 tumor-bearing mice. We hypothesized that the superior in vivo stability of AMTG in combination with ^161^Tb would result in improved tumor control and overall survival as compared to RM2 and ^177^Lu counterparts.

**Methods:**

Treatment studies in PC-3 tumor-bearing Nu/J mice were initiated once tumor volume was ~ 100 mm^3^. ^161^Tb- and ^177^Lu-Labeling was completed at 90 °C within 10 min (1.0 M sodium acetate buffer, pH = 5.5, molar activity of ~ 50 MBq/nmol). Radiolabeled GRPR ligands were administered in treatment (PC-3 tumor-bearing, n = 6–7 per group) and toxicity (healthy animals, n = 3 per group) animals on day 0 and day 7 of the experiment (~ 15 MBq each). Treatment animals were sacrificed once tumor volume surpassed 1,500 mm^3^. Toxicity animals were sacrificed 45 d after injection and analyzed for complete blood count and metabolic panel.

**Results:**

Animals were assigned to five groups (control, [^177^Lu]Lu-RM2, [^177^Lu]Lu-AMTG, [^161^Tb]Tb-RM2, [^161^Tb]Tb-AMTG). Each treatment group received a total activity amount of 26–31 MBq of the respective radiolabeled compound. All treatments resulted in improved tumor control and overall survival related to vehicle animals. [^161^Tb]Tb-AMTG had the longest median overall survival of 58.1 ± 5.4 d after initiation of treatment. No signs of long-term toxicity were observed during this study in any treatment group.

**Conclusion:**

The combination of Auger electrons with high in vivo stability of [^161^Tb]Tb-AMTG resulted in substantially improved tumor control and overall survival in PC-3 tumor-bearing mice, thus expanding the therapeutic potential of GRPR antagonists in GRPR-expressing malignancies.

**Graphical Abstract:**

**Figure:**
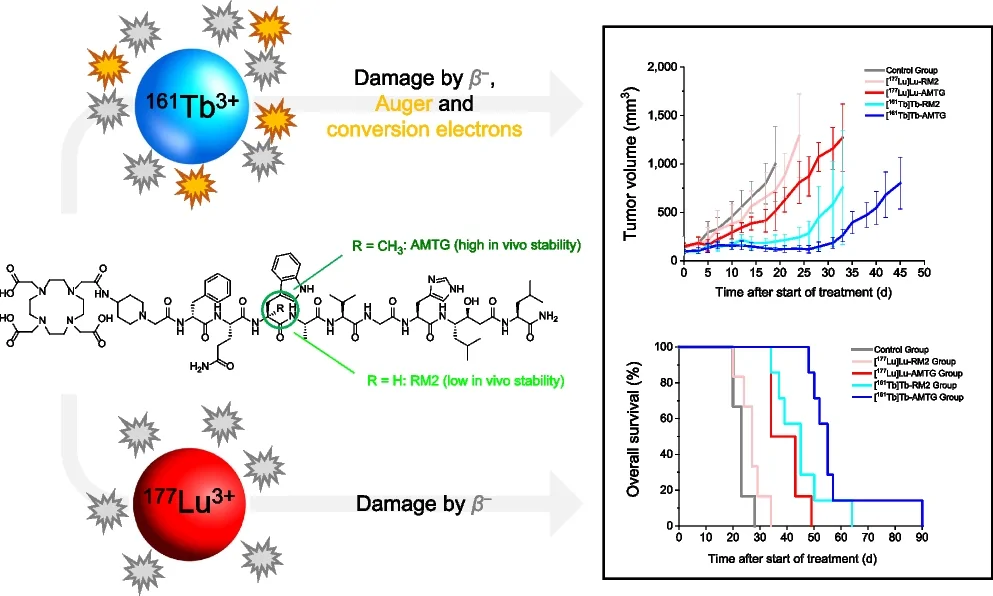

**Supplementary Information:**

The online version contains supplementary material available at https://doi.org/10.1007/s00259-026-08035-6.

## Introduction

Over the last decade, the β particle emitter lutetium-177 (^177^Lu) has been established as the predominant therapeutic radiometal in nuclear medicine. Due to its favorable physical properties (E_*β*−,mean_ ~ 0.14 MeV, *t*_1/2_ = 6.647 d [1]), it is the main radioisotope currently used in clinical trials as well as the two FDA-approved compounds, [^177^Lu]Lu-PSMA-617 (Pluvicto^®^) and [^177^Lu]Lu-DOTATATE (Lutathera^®^) [2]. Despite the success of radiopharmaceutical therapy (RPT) using ^177^Lu, stronger particles like α-emitters such as actinium-225 (^225^Ac), lead-212 (^212^Pb) or astatine-211 (^211^At) [3–5] or Auger electron emitting radionuclides such as terbium-161 (^161^Tb, E_*β*−,mean_ ~ 0.15 MeV, *t*_1/2_ = 6.90 d) [1] are currently experiencing noticeable attention. The improved efficacy of α particles and Auger electrons is associated with their significantly higher linear energy transfer compared with β particles (50–230 and 4–26 versus 0.1–1.0 keV/µm, respectively) [6]. An advantage of ^161^Tb over α-emitters represents that like ^177^Lu it forms stable complexes with the clinically established chelator 2,2′,2′′,2′′′-(1,4,7,10-Tetraazacyclododecane-1,4,7,10-tetrayl)tetraacetic acid (DOTA), which allows for labeling of the same radiopharmaceutical precursors already in clinical use for ^177^Lu-based RPT [7].

Owing to the additional emission of Auger and conversion electrons (on top of β particle emissions), resulting in an increased dose deposited at the target site [8], a substantially higher dose response [9] and treatment efficacy [10] was observed for a ^161^Tb-labeled somatostatin-2 receptor antagonist compared with its ^177^Lu-analog or a ^161^Tb-labeled somatostatin-2 receptor agonist. This suggests cell membrane damage caused by short-ranged Auger electrons (‘membrane effect’, exact mechanism unclear) [11, 12], which is contradictory to the belief that Auger electrons must be close to the cell nucleus to damage (tumor) cells. Increased treatment efficacy was also observed for the prostate-specific membrane antigen inhibitor [^161^Tb]Tb-PSMA-617 in PC-3 Pip tumor-bearing mice when compared with its ^177^Lu-analog [13], as well as for the folate receptor targeted compound [^161^Tb]Tb-cm09 in KB tumor-bearing mice when compared with its ^177^Lu-derivative [14]. However, no significant difference in survival was observed for the fibroblast activation protein inhibitor FAP-2286 in a preclinical pancreatic ductal adenocarcinoma model when labeled with ^177^Lu or ^161^Tb [15], suggesting that the beneficial ‘membrane effect’ might be dependent on the properites of the target.

In order to extend the ^161^Tb versus ^177^Lu comparison we previously reported on a preclinical study investigating gastrin-releasing peptide receptor (GRPR) antagonists [16]. While several GRPR ligands have already proven clinical value for positron emission tomography imaging, successful therapeutic applications of radiolabeled GRPR ligands are scarce to date [17]. Apart from the physical properties of the applied radionuclides, sufficient in vivo stability of the target vector, which is a known issue of GRPR-targeted compounds, is also an important factor for successful RPT [18]. While the GRPR antagonist [^177^Lu]Lu-RM2 revealed promising clinical data [19], its pharmacophore-modified analog, [^177^Lu]Lu-AMTG, exhibited improved in vivo stability in mice [20] and humans [21], which may potentially lead to improved treatment efficacy. Due to favorable dosimetry calculated for [^177^Lu]Lu-AMTG in a small cohort of prostate cancer patients [22] and based on the favorable biodistribution profile of [^161^Tb]Tb-AMTG observed preclinically [16], we consider the investigation of the therapeutic efficacy of RM2 and AMTG analogs a logical progression of our previous work which is scientifically necessary with regard to a potential clinical translation of ^161^Tb-labeled GRPR ligands.

Therefore, in this study we completed a comparative preclinical evaluation of [^161^Tb]Tb-/[^177^Lu]Lu-AMTG and [^161^Tb]Tb-/[^177^Lu]Lu-RM2 with respect to treatment efficacy and potential long-term toxicity in PC-3 tumor-bearing (treatment) and healthy (toxicity) mice.

## Materials and methods

### Synthesis and radiolabeling

Precursor synthesis and ^161^Tb-/^177^Lu-labeling was performed according to a published procedure [20]. ^161^Tb was provided by the PRISMAP project, produced by irradiation of enriched ^160^Gd targets in the high flux reactors BR2 (SCK CEN, Mol, Belgium) and RHF (ILL, Grenoble, France), and radiochemically separated at SCK CEN. [^177^Lu]LuCl_3_ was acquired from ITM Isotope Technologies Munich SE (Munich, Germany).

### Treatment and toxicity studies

Animal experiments were carried out according to protocols approved by the Institutional Animal Care and Use Committee at Stanford University and were performed based on the NIH Guide for the Care and Use of Laboratory Animals. Nu/J mice aged 6–8 weeks (The Jackson Laboratory, Sacramento, CA, USA) were used. After arrival at the in-house facilities, mice were allowed to acclimate for a minimum of one week before inoculation of PC-3 cells (human prostate cancer cell line; isolated from a bone marrow metastasis in a Caucasian suffering from advanced prostate cancer) according to a previously reported protocol [20]. Animals were excluded from the study when reaching one of the following endpoints: > 20% weight loss, tumor ulceration, respiratory distress, or change of behavior. None of these criteria applied to any animal from the experiment. All control or treatment animals were sacrificed once a tumor volume of greater than 1,500 mm^3^ was reached, or 90 days after initiation of the experiment.

To establish tumor xenografts, PC-3 (CRL-1435, ATCC, Manassas, VA, USA) cells (5.0 × 10^6^ cells per 200 µL, handling described in the supplementary information) were suspended in a 1/1 mixture (*v*/*v*) of phosphate-buffered saline solution and Cultrex Basement Membrane Extract, Type 3, Pathclear (R&D Systems™, Minneapolis, MN, USA) and inoculated subcutaneously onto the right shoulder of the animals. Mice were used for treatment studies (^177^Lu groups and control group: n = 6, ^161^Tb groups: n = 7) once a tumor volume of 100–150 mm^3^ (2–3 weeks after inoculation) was reached.

Approximately 15 MBq (300 pmol) of the ^161^Tb- or ^177^Lu-labeled GRPR ligand were injected into the tail vein of anesthetized (2% isoflurane) PC-3 tumor-bearing mice at days 0 and 7 of the experiment. Animals were monitored for up to 90 days three times per week. Tumors were measured using a caliper, and tumor volume was calculated according to the formula V = 0.5 × (small diameter)^2^ × (long diameter).

Toxicity animals (healthy animals, each group n = 3) were sacrificed 45 days after initiation of the experiment. Blood (~ 1 mL per animal) was taken via cardiac puncture, and approximately 200 µL (per animal) were transferred into dipotassium-EDTA tubes (BD Microtainer™ Capillary Blood Collector and BD Microgard™ Closure, Lavender, Thermo Fisher Scientific Inc., Waltham, MA, USA) for determination of complete blood count. The remaining blood was transferred into Serum Separator Tubes (BD Microtainer™ Capillary Blood Collector and BD Microgard™ Closure, Gold, Beckton Dickinson, NJ, USA), centrifuged at 10,000 × g for 2 min, and serum was separated subsequently. Serum parameters were determined by the Animal Diagnostic Lab at Stanford University (in-house, Stanford, CA, USA). Kidneys and pancreas of each animal was excited and placed in 4% paraformaldehyde. Tissue embedding, and hematoxylin and eosin staining was done by Histo-Tec Laboratory Inc. (Hayward, CA, USA). Light microscopy images were obtained using a Hamamatsu Photonics NanoZoomer (Hamamatsu, Shizuoka, Japan).

### Statistics

Acquired data were statistically analyzed either by using a Student’s *t-*test (tumor volume) using OriginPro software (version 9.7) from OriginLab Corporation (Northampton, MA, USA), or a Logrank test (survival), as well as a two-way analysis of variance (ANOVA) test and a Tukey’s multiple comparisons test (blood parameters) using the GraphPad Prism 11 software (GraphPad Software Inc*.*, San Diego, CA, USA). Acquired *p* values of < 0.05 were considered statistically significant.

## Results

### Radiolabeling

^161^Tb- and ^177^Lu-Labeling resulted in radiochemical yields and purities of > 98% and apparent molar activities of 50 ± 2 GBq/μmol. All ^161^Tb- and ^177^Lu-labeled compounds were used without further purification.

### Treatment studies

Animals were assigned to five groups (average tumor volume; control: 139 ± 36 mm^3^, [^177^Lu]Lu-RM2: 156 ± 88 mm^3^, [^177^Lu]Lu-AMTG: 150 ± 34 mm^3^, [^161^Tb]Tb-RM2: 98 ± 22 mm^3^, [^161^Tb]Tb-AMTG: 101 ± 21 mm^3^) at day 0 of the treatment study. Radioactivity was administered on days 0 and 7, while each treatment group received a total activity amount of 26–31 MBq of the respective radiolabeled GRPR ligand ([^177^Lu]Lu-RM2: 31.0 ± 1.2 MBq, [^177^Lu]Lu-AMTG: 26.0 ± 2.8 MBq, [^161^Tb]Tb-RM2: 30.3 ± 0.7 MBq, [^161^Tb]Tb-AMTG: 29.0 ± 1.2 MBq).

**Fig. 1:**
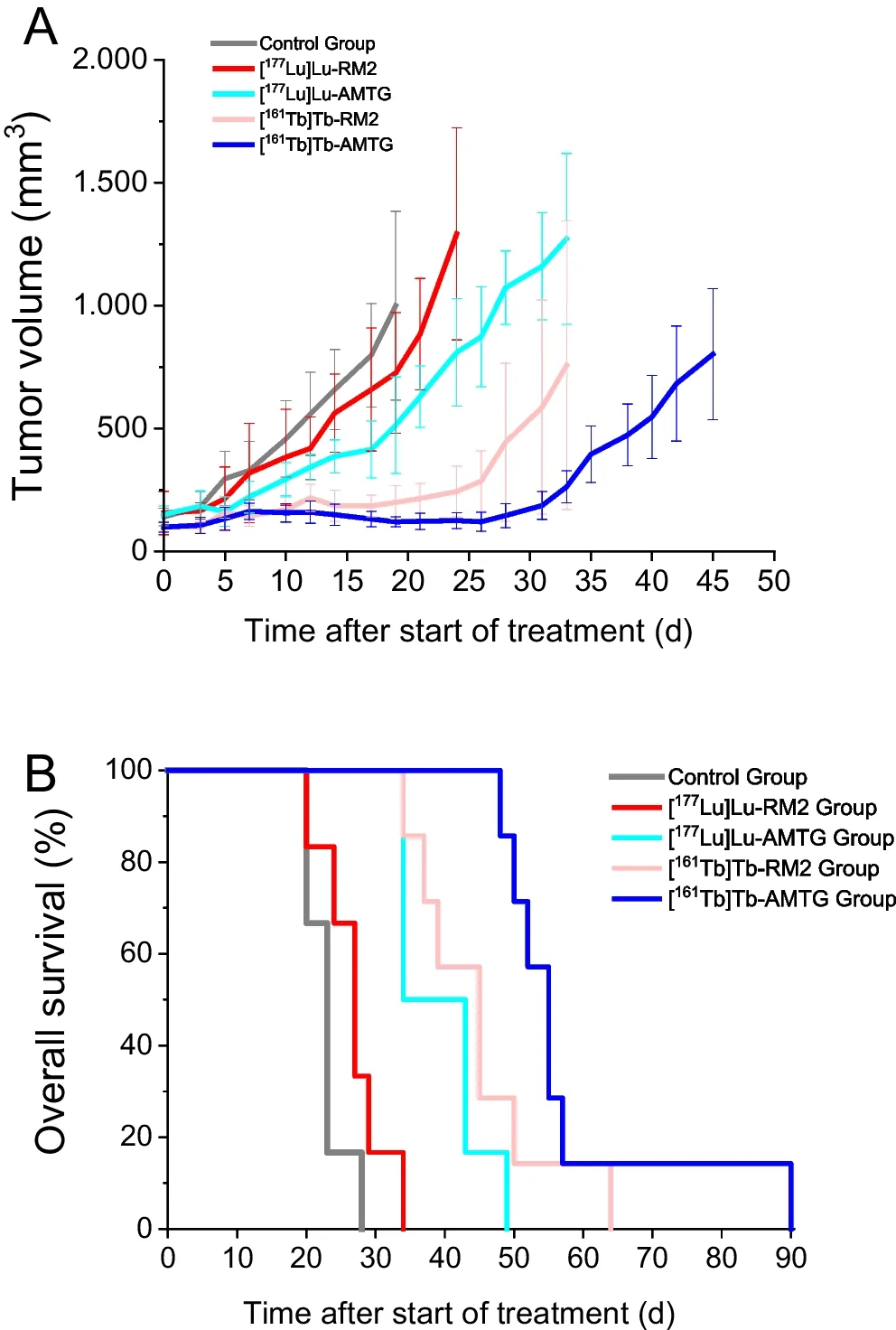
Treatment efficacy of ^177^Lu- and ^161^Tb-labeled GRPR ligands in PC-3 tumor-bearing Nu/J mice depicted by their impact on (**A**) tumor growth (expressed as mean volume ± SD) and (**B**) overall survival (Kaplan–Meier curve). Radioactivity (in total; [^177^Lu]Lu-RM2: 31.0 ± 1.2 MBq, [^177^Lu]Lu-AMTG: 26.0 ± 2.8 MBq, [^161^Tb]Tb-RM2: 30.3 ± 0.7 MBq, [^161^Tb]Tb-AMTG: 29.0 ± 1.2 MBq) was administered on days 0 and 7

Compared with the control group which did not receive any radioactivity injection, all treatment groups achieved an improved tumor control over time (Fig. 1A). At day 19, the control group comprised an average tumor volume of 999 ± 384 mm^3^, while all treatment groups displayed a lower average tumor volume ([^177^Lu]Lu-RM2: 725 ± 246 mm^3^, [^177^Lu]Lu-AMTG: 513 ± 196 mm^3^, [^161^Tb]Tb-RM2: 199 ± 68 mm^3^, [^161^Tb]Tb-AMTG: 119 ± 20 mm^3^). This indicates a significantly reduced tumor growth of the [^161^Tb]Tb-AMTG group compared to [^161^Tb]Tb-RM2 (*p* < 0.01) as well as compared with the ^177^Lu-groups (*p* < 0.001) and the control group (*p* < 0.0001). Significant differences in tumor growth were also observed for [^161^Tb]Tb-RM2 when compared with the ^177^Lu-groups (*p* < 0.001) and the control group (*p* < 0.0001). Treatment with [^177^Lu]Lu-AMTG led to significantly reduced tumor growth compared with the control group (*p* < 0.05), while no statistically significant difference was determined between the [^177^Lu]Lu-AMTG group and the [^177^Lu]Lu-RM2 group (*p* = 0.07), as well as between the [^177^Lu]Lu-RM2 group and the control group (*p* = 0.11). At day 33, the remaining treatment groups showed noticeable differences in average tumor volume, with [^161^Tb]Tb-AMTG (262 ± 65 mm^3^) revealing the most effective tumor control, followed by [^161^Tb]Tb-RM2 (756 ± 587 mm^3^, *p* = 0.02) and [^177^Lu]Lu-AMTG (1271 ± 348 mm^3^, *p* < 0.0001). A statistically significant difference in tumor growth was also observed between the [^161^Tb]Tb-RM2 and the [^177^Lu]Lu-AMTG group (*p* = 0.044).

**Fig. 2:**
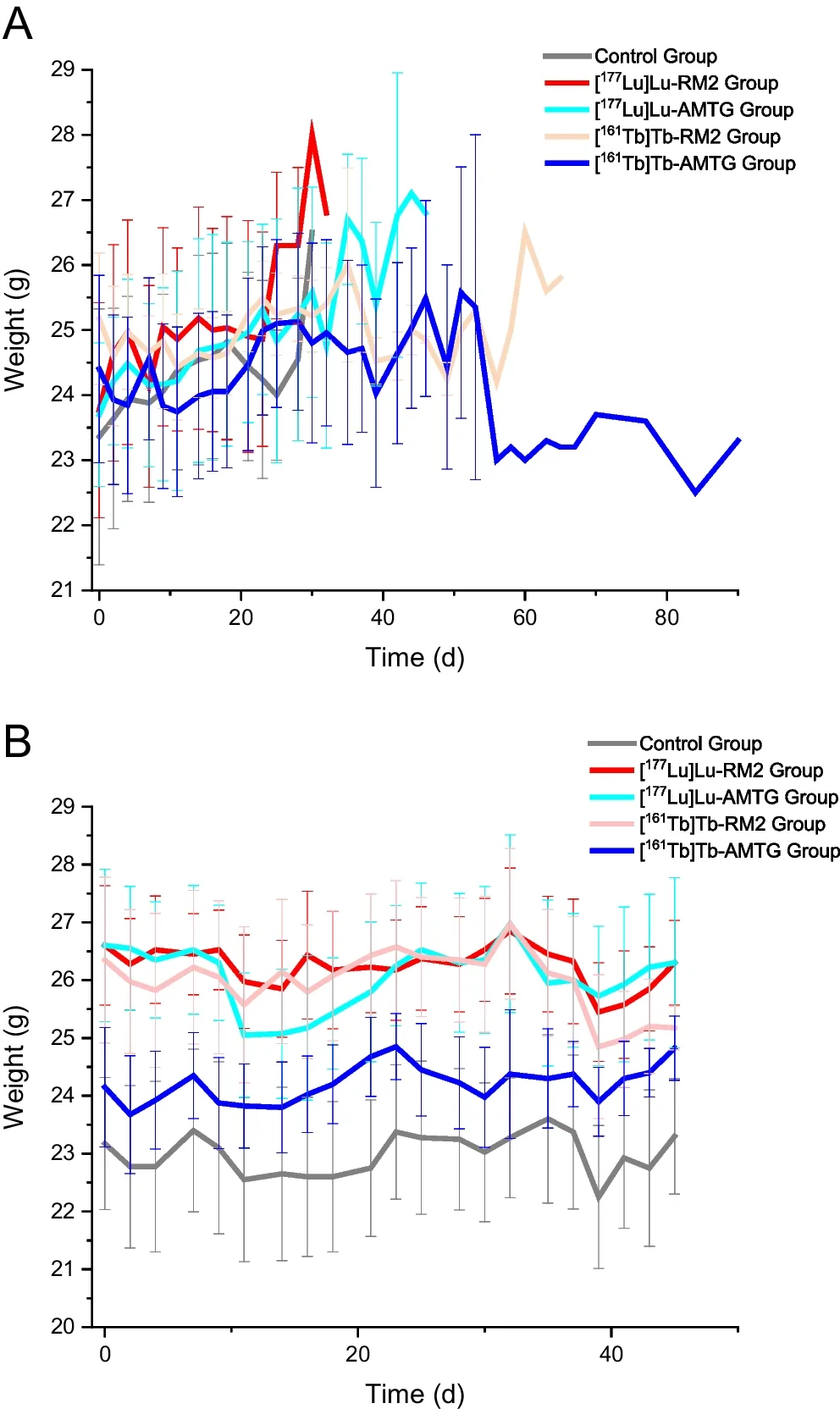
Total body weight of (**A**) control and treatment (mean, n = 6–7) and (**B**) toxicity animals (mean, n = 3) over time

Overall survival after initiation of treatment revealed significant differences between the groups (*p* < 0.0001), as survival was best when tumor-bearing mice were treated with [^161^Tb]Tb-AMTG (58.1 ± 5.4 d), followed by [^161^Tb]Tb-RM2 (44.9 ± 3.8 d), [^177^Lu]Lu-AMTG (39.5 ± 2.6 d), and [^177^Lu]Lu-RM2 (26.8 ± 1.9 d), while it was worst for the non-treated control group (22.8 ± 1.2 d) (Fig. 1B). Pairwise comparison of survival curves demonstrated statistical significance for the control group versus the [^177^Lu]Lu-AMTG group (*p* = 0.0006), the [^161^Tb]Tb-RM2 group (*p* = 0.0003), and the [^161^Tb]Tb-AMTG group (*p* = 0.0003), while it was not statistically significant versus the [^177^Lu]Lu-RM2 group (*p* = 0.089). Moreover, statistical significance was found for the [^177^Lu]Lu-RM2 group versus the [^177^Lu]Lu-AMTG group (*p* = 0.0033), the [^161^Tb]Tb-RM2 group (*p* = 0.0005), and the [^161^Tb]Tb-AMTG group (*p* = 0.0002). While statistical significance was obtained for the [^177^Lu]Lu-AMTG group versus the [^161^Tb]Tb-AMTG group (*p* = 0.0007), it was not obtained versus the [^161^Tb]Tb-RM2 group (*p* = 0.20). No statistical significance was achieved for the [^161^Tb]Tb-RM2 group versus the [^161^Tb]Tb-AMTG group (*p* = 0.08). On average, all animals gained weight over the course of the experiment (Fig. 2A).

### Toxicity studies

In general, no noticeable changes in weight were observed for the control group or the treatment groups (Fig. 2B).

**Fig. 3:**
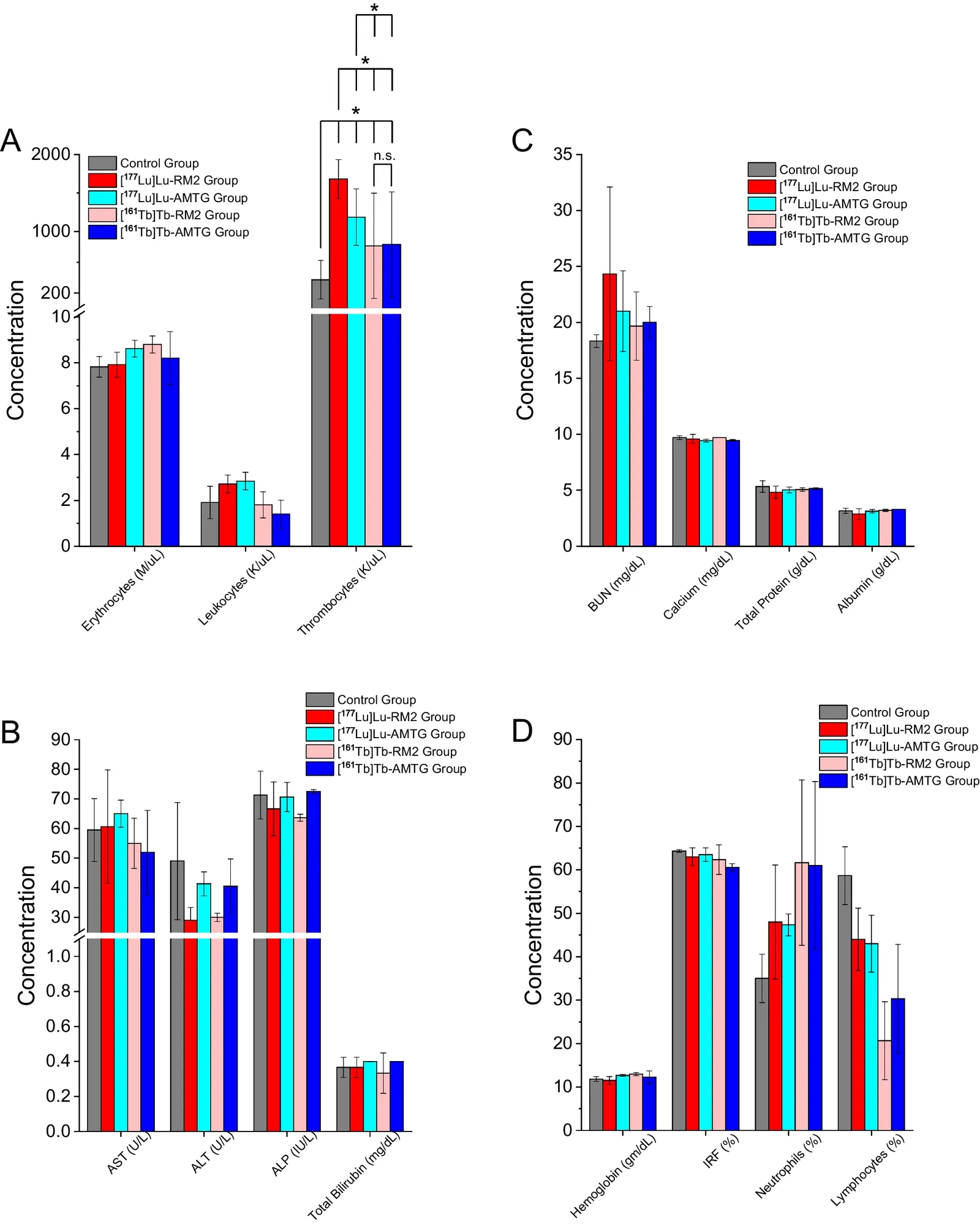
Serum parameters of healthy Nu/J mice sacrificed at 45 d after injection of [^177^Lu]Lu-RM2, [^177^Lu]Lu-AMTG, [^161^Tb]Tb-RM2, and [^161^Tb]Tb-AMTG, as well as of the control group (no radioactivity received). Data expressed as mean ± SD. Concentration of (A) erythrocytes, leukocytes, and thrombocytes; (B) aspartate aminotransferase (AST), alanine aminotransferase (ALT), alkaline phosphatase (ALP), and total bilirubin [to check for potential liver toxicity]; (C) blood urea nitrogen (BUN), calcium, total protein, and albumin [to check for potential kidney toxicity]; (D) hemoglobin, immature reticulocyte fraction (IRF), neutrophils, and lymphocytes

Analysis of complete blood count, as well as chemistry and electrolyte profiles did not show significant differences between the control and treatment groups (Fig. 3A-D, Supplemental Fig. 1), thus not indicating treatment-related long-term toxicity or malnutrition (Fig. 3C). A difference in the thrombocyte concentration between each group was statistically significant (*p* < 0.01, Fig. 3A), except when comparing the [^161^Tb]Tb-AMTG and [^161^Tb]Tb-RM2 groups (*p* = 0.99). Noteworthy, animals treated with ^161^Tb-labeled GRPR ligands exhibited a lower lymphocyte concentration than animals treated with their ^177^Lu-labeled analogs, and all treatment group animals revealed a lower lymphocyte concentration than the untreated control animals, however, this was not statistically significant (*p* > 0.95, Fig. 3D).

**Fig. 4:**
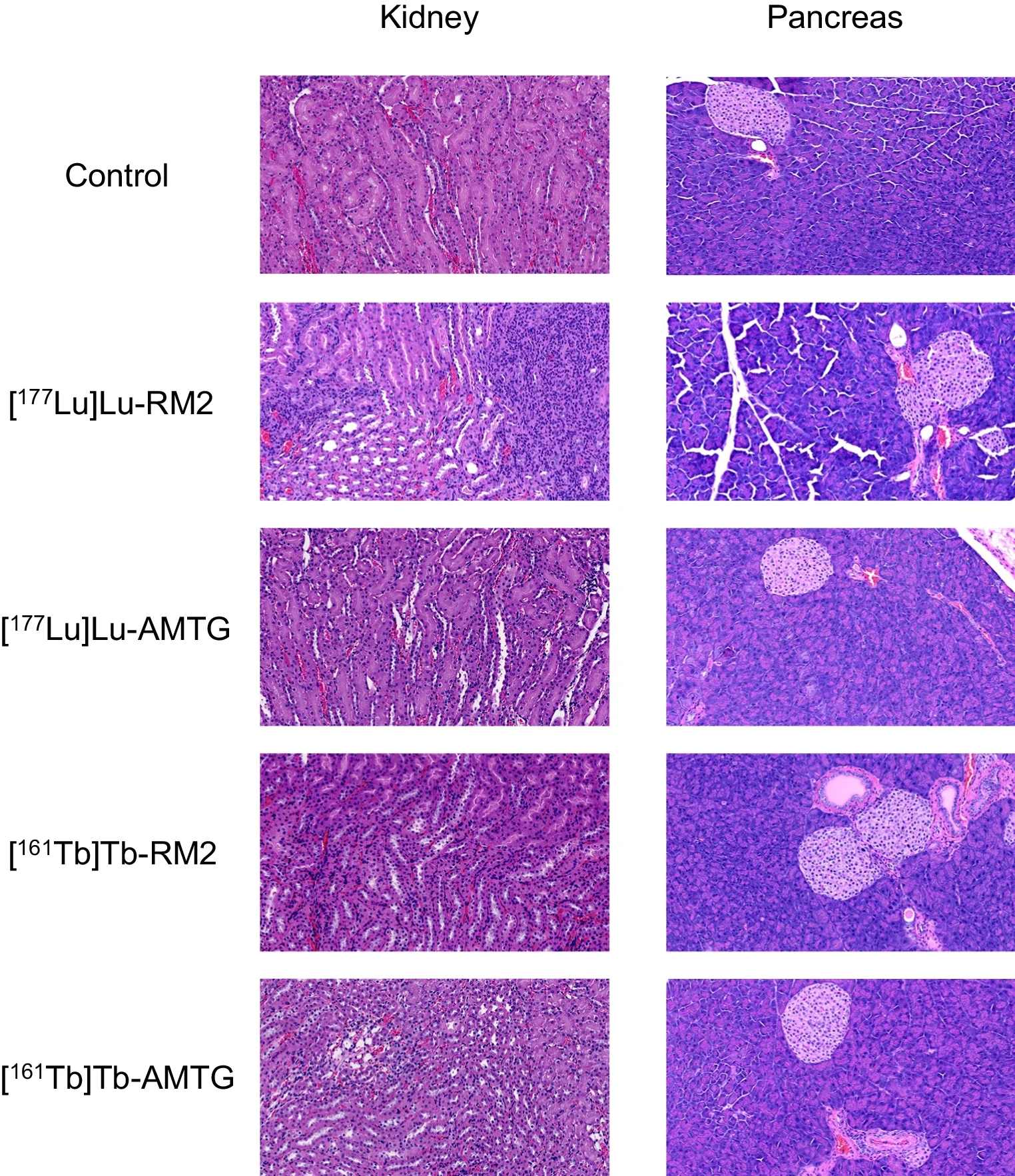
Representative light microscopy images (20 × each) of hematoxylin and eosin-stained kidney and pancreas tissue sections of healthy Nu/J mice sacrificed at 45 d after injection of [^177^Lu]Lu-RM2, [^177^Lu]Lu-AMTG, [^161^Tb]Tb-RM2, or [^161^Tb]Tb-AMTG, as well as of the control group (no radioactivity received)

Histopathologic comparison of pancreatic and renal tissue of the control group with the different treatment groups did not show any indication of long-term toxicity (Fig. 4).

## Discussion

In the field of nuclear medicine, *β*-particle emitting radionuclides such as ^177^Lu currently build the foundation of RPT, while *α*-particle emitting radionuclides such as ^225^Ac, ^212^Pb, and ^211^At are on the rise [4, 5]. Apart from these particles, the recent discovery of a possible combination of antagonistic peptide structures with short-ranged Auger electron emitting radionuclides such as ^161^Tb [10] might extend the toolbox of available RPT strategies. Therefore, we evaluated the treatment efficacy of ^161^ Tb-labeled GRPR antagonists compared with their ^177^Lu-labeled analogs in PC-3 tumor-bearing mice.

Radiolabeling proceeded quantitatively for ^177^Lu and ^161^Tb using a previously published protocol [20], achieving high molar activities of 50 GBq/µmol. Interestingly, before we started the treatment and toxicity studies, we encountered some difficulties with regard to ^161^Tb-labeling, as using the same protocol only proceeded quantitatively when a higher amount of precursor was used, which resulted in a significantly lower molar activity (20 GBq/µmol). This was unexpected, as previous work using this protocol resulted in comparable molar activities using ^177^Lu and ^161^Tb for labeling of RM2 and AMTG [16]. Intriguingly, though the same reagents and radiolabeling precursor solutions were used for both ^177^Lu- and ^161^Tb-labeling, radiochemical yields and molar activities were significantly higher when using ^177^Lu. Only the preparation of a fresh batch of each precursor and buffer solution avoiding any contact to metals (for the previous batches, a metal spatula was used to weigh in the reagents) led to quantitative yields and similarly high molar activities for the ^161^Tb-labeled compounds. Even though different behavior of ^177^Lu and ^161^Tb had not been observed in a comparative evaluation [7], we found that ^161^Tb-labeling was more sensitive than ^177^Lu-labeling, and while the latter seemed to tolerate trace contaminations potentially caused by using a metal spatula, the former was more susceptible towards this. Therefore, all ^161^Tb-labelings in this work were completed using the freshly prepared buffer solutions, which is why the ^161^Tb-labeled compounds were administered at an apparent molar activity of 50 GBq/µmol.

All treatment groups received a similar total activity of approximately 30 MBq via fractionated dosing (two doses of 15 MBq each, seven days apart from each other). A similar dosing regimen was used by Borgna et al. for their ^161^Tb-/^177^Lu-labeled somatostatin analogs (two doses of 10 MBq each, seven days apart from each other) [10]. Small discrepancies in total activity administered between RM2 and AMTG were caused by the slightly higher lipophilicity of the latter, which led to slightly more activity retained in the syringe and catheter.

Both ^161^Tb-labeled GRPR ligands revealed improved tumor control and overall survival compared with their ^177^Lu-counterparts, which indicates a benefit of additionally emitted Auger electrons on treatment efficacy, similar to what was observed by Borgna et al., when the respective radionuclide (e.g., ^161^Tb) was attached to the predominantly membrane-bound somatostatin-2 receptor antagonists [10]. An additional factor that could explain the improved therapeutic efficacy of ^161^Tb-labeled compounds includes our prior demonstration of the higher biological half-life for the ^161^Tb-labeled compounds over their ^177^Lu-labeled analogs [16]. Despite being retained significantly longer at the tumor due to its increased in vivo stability [16], [^177^Lu]Lu-AMTG displayed slightly less tumor control than did [^161^Tb]Tb-RM2, further suggesting the impact of Auger electrons on tumor damage, even though the difference in survival was not statistically significant. As expected, treatment with [^161^Tb]Tb-AMTG led to the most effective tumor control and overall survival, with one animal showing complete remission, which can be associated with its high in vivo stability combined with the additional Auger and conversion electron emissions. On average, AMTG led to an approximately 14 d longer survival than RM2 when labeled with the same radionuclide, which highlights the importance of high in vivo stability [21, 22]. Although median survival of the [^161^Tb]Tb-AMTG (58.1 ± 5.4 d) and the [^161^Tb]Tb-RM2 (44.9 ± 3.8 d) group was not statistically significant (*p* = 0.08), statistical significance was obtained when excluding the respective outliers ([^161^Tb]Tb-RM2: one mouse survived 64 d, [^161^Tb]Tb-AMTG: one mouse survived 90 d) of each group (*p* = 0.004, median survival of 52.8 ± 1.4 d [[^161^Tb]Tb-AMTG] and 41.7 ± 2.4 d [[^161^Tb]Tb-RM2]). Surprisingly, treatment with two doses of 15 MBq each of [^177^Lu]Lu-RM2 did not result in a statistically significant difference in survival compared to the untreated group. Hence, future studies that aim to optimize the therapeutic efficacy of [^177^Lu]Lu-RM2 could investigate higher doses or increased fractionation.

A recently reported study on [^47^Sc]Sc-LF1 ([^47^Sc]Sc-AAZTA^5^-Pip-D-Phe-Gln-Trp-Ala-Val-Gly-His-Sta-Leu-NH_2_) revealed an overall survival of ~ 27 d and ~ 32 d in PC-3 tumor-bearing mice using 30 MBq (three doses of 10 MBq each on day 0, 2, and 4) and 60 MBq (six doses of 10 MBq each on day 0, 2, 4, 13, 15, and 17), respectively [23]. This was lower than the overall survival achieved with [^161^Tb]Tb-AMTG (~ 58 d), [^161^Tb]Tb-RM2 (~ 45 d) and [^177^Lu]Lu-AMTG (~ 40 d), but higher or comparable to that achieved with [^177^Lu]Lu-RM2 (~ 27 d). In another study, treatment of PC-3 tumor-bearing mice using a cumulative activity of ~ 41 MBq (six doses of ~ 7 MBq each on day 0, 2, 4, 13, 15, and 17) of [^177^Lu]Lu-LF1 ([^177^Lu]Lu-AAZTA^5^-Pip-D-Phe-Gln-Trp-Ala-Val-Gly-His-Sta-Leu-NH_2_) led to an overall survival of ~ 25 d, while treatment using ~ 93 MBq of [^177^Lu]Lu-LF1 (six doses of ~ 15.5 MBq each on day 0, 2, 4, 13, 15, and 17) increased overall survival to ~ 50 d [24]. Although total activities injected were equal (30 MBq) or higher (41 MBq, 60 MBq and 93 MBq, respectively), the dose regimens were different, with our compounds administered only twice (~ 15 MBq each, day 0 and day 7). Even though dose regimens were slightly different, and comparison of data generated by different groups in different mouse strains (Nu/J versus Balb/C nude mice) have to be considered with caution, these results further indicate the effect of both high in vivo stability of the peptide and the radionuclide used.

In general, our findings corroborate with previous studies, as a similar biodistribution was observed for ^161^Tb- and ^177^Lu-labeled RM2 and AMTG, yet a higher activity uptake over time (area under the curve) was found in the tumor for the ^161^Tb- compared with their respective ^177^Lu-labeled analogs [16]. Notably, despite higher areas under the curve observed for [^177^Lu]Lu-/[^161^Tb]Tb-AMTG particularly in the pancreas which endogenously expresses GRPR and in the kidneys due to renal clearance [16], both representing organs at risk, no signs of long-term toxicity were observed in hematoxylin and eosin-stained kidney and pancreas tissue sections. Moreover, serum parameters that would indicate either liver (Fig. 3B) or kidney (Fig. 3C) toxicity were unremarkable between the control and the treatment groups, which confirmed that the dosing regimen applied (two doses of 15 MBq each of ^177^Lu- or ^161^Tb-labeled RM2 or AMTG) did not appear to harm healthy organs while it significantly damaged the tumor xenograft. This is in line with previous studies that demonstrated a total activity of up to 144 MBq (6 × 24 MBq) of [^177^Lu]Lu-RM2 did not result in treatment-related toxicity, while good to high treatment efficacy was observed [25].

In general, no substantial changes in body weight were observed for the toxicity animals, and a slight increase in weight was observed for both the control group and the treatment groups over the course of the study, which indicates no significant toxicity for either of the radiopharmaceuticals administered. However, it has to be mentioned that growing tumor xenografts in treatment animals also slightly contributed to the overall weight (tumor weight at endpoint lower than 1.5 g). Furthermore, the only serum parameter that was significantly different between the control group and the treatment groups represented the thrombocyte concentration. This was not anticipated, as radiotherapy usually leads to a decrease in thrombocytes, not an increase [26, 27]. A potential explanation for this reactive thrombocytosis could be an infection, an inflammation, the tumor burden, or a hyperactive bone marrow that temporarily overproduces platelets while it is recovering from treatment [28]. Therefore, in-depth toxicity studies would be necessary to further explore the observed thrombocytosis, which was not the focus of this study, as we only aimed at the comparison between ^177^Lu- and ^161^Tb-labeled AMTG and RM2, and the elimination of any gross toxicity associated with these agents. Although not significant, most likely due to the low animal number used (n = 3 per group), a difference in the lymphocyte concentration was observed. While all treatment animals demonstrated a slightly lower lymphocyte level compared with the control group, a lower lymphocyte count was observed in animals treated with ^161^Tb- compared to ^177^Lu-labeled GRPR ligands, which was expected, as lymphopenia is frequently observed after RPT [27]. This was also reported for ^161^Tb-labeled somatostatin analogs [29], and can be attributed to the additional emission of Auger and conversion electrons. As lymphocytes eventually recover over time [29], careful management of treatment cycles can usually prevent severe lymphopenia. Preliminary results from the VIOLET trial ([^161^Tb]Tb-PSMA I&T) revealed that grade 3 lymphopenia was observed in only 3% of patients after receiving up to six cycles of 7.4 GBq each [30].

A limitation of this study represents the low animal number used per group for the toxicity studies, which only permits detection of gross toxicity and which is why toxicity data obtained in this study has to be considered with caution. Furthermore, only long-term toxicity was tested to ensure that no treatment-related adverse events were detected in animals that showed improved overall survival. As the focus of this work was to compare the overall efficacy and long-term toxicity of AMTG and RM2 ligands labeled with ^177^Lu or ^161^Tb, an extensive toxicity study that encompassed multiple timepoints post radiotherapeutic administration was beyond the scope of this work. Furthermore, in order to obtain toxicity data in relation to T-lymphocytes, future toxicity studies should be assessed in immunocompetent mice. Although our study does not assess treatment-associated toxicity at early timepoints post treatment initiation, the lack of observable toxicity at 45 days post treatment administration suggests either the recovery of any early toxicity or lack of toxicity on the parameters studied. Another limitation is the injection of only two doses of the respective radiopharmaceutical, which is not representative of the clinical situation, as usually up to six cycles are administered. More doses would have likely resulted in improved tumor control and overall survival, as 6 × 6 MBq of [^177^Lu]Lu-RM2 (Dumont et al. [25]) led to substantially higher mean survival than 2 × 15 MBq of [^177^Lu]Lu-RM2 in our study (42 d versus 27 d). However, due to the limited availability of ^161^Tb, and for comparative reasons, we elected to apply a dose regimen of two injections of 15 MBq each of the respective ^177^Lu- and ^161^Tb-labeled compounds.

Overall, this study further highlights the importance of high in vivo stability, but also indicates that the ‘membrane effect’ observed preclinically for somatostatin-2 receptor antagonists might also be present for GRPR antagonists. Based on previously determined dosimetry data on [^177^Lu]Lu-AMTG, which showed a dose rate of 3.1 ± 0.8 and 1.8 ± 0.9 Gy/GBq in osseous and soft tissue lesions, respectively, the use of [^161^Tb]Tb-AMTG could result in dose rates of ~ 4.3 and 2.5 Gy/GBq, respectively, when assuming a dose enhancement factor of 1.4 reported by Verburg et al. for DOTATATE and PSMA-617 labeled with ^161^Tb instead of ^177^Lu [31]. Applying a dose enhancement factor of 2.4 observed by Schaefer-Schuler et al. during clinical comparison of [^161^Tb]Tb-PSMA-617 and its ^177^Lu-analog [8], a dose rate of ~ 7.4 and 4.3 Gy/GBq in osseous and soft tissue lesions, respectively, might be achievable for [^161^Tb]Tb-AMTG. However, this remains speculative and future studies, particularly in cancer patients, are required to elucidate whether the application of antagonists labeled with radionuclides emitting short-ranged Auger electrons or α-particles can improve clinical outcomes.

## Conclusion

Combination of the more powerful radionuclide ^161^Tb with the metabolically more stable GRPR ligand AMTG led to improved treatment efficacy in PC-3 tumor-bearing mice. Both the radionuclide (^161^Tb > ^177^Lu) and the precursor (AMTG > RM2) applied each had a substantial impact on tumor control and overall survival, while treatment-related long-term toxicity was not observed. Based on these preclinical results paired with our experiences with [^177^Lu]Lu-AMTG in a clinical setting, clinical translation of [^161^Tb]Tb-AMTG is feasible.

## Supplementary Information

Below is the link to the electronic supplementary material.

Supplementary file1 (DOCX 89.1 KB)

## Data Availability

The datasets generated during and/or analysed during the current study are available from the corresponding author on reasonable request.
