# Supplementary material for: Evaluation of treatment efficacy of the 161Tb- and 177Lu-Labeled GRPR antagonists AMTG and RM2 in a mouse model of prostate cancer
Source: Eur J Nucl Med Mol Imaging. 2026 Jul 10;53(12):7006–16. doi: 10.1007/s00259-026-08035-6 (PMC13633373; doi:10.1007/s00259-026-08035-6)
Supplement: Supplementary file 1 [file 259_2026_8035_MOESM1_ESM.docx]

**– Supplementary Material –**

Thomas Günther^1,2,^*, Abraham S Moses^1^, Elisabeth Günther^1^, Madeleine R Landry^1^, Stijn Ramaekers^3^, Michiel Van de Voorde^3^, Maarten Ooms^3^, Bernard Ponsard^4^, Corinne Beinat^1^

^1^ Molecular Imaging Program at Stanford (MIPS), Department of Radiology, School of Medicine, Stanford University, Stanford, CA, USA

^2^ University Duisburg-Essen and DKTK partner site University Hospital Essen, Department of Nuclear Medicine, Essen, Germany

^3^ Nuclear Medical Applications, Belgian Nuclear Research Centre, Mol, Belgium

^4^ BR2 Reactor, Belgian Nuclear Research Centre, Mol, Belgium

***Corresponding author:**

Thomas Günther, PhD

[tgunther@stanford.edu](mailto:tgunther@stanford.edu); thomas.guenther@uk-essen.de

ORCID-ID: [0000-0002-7412-0297](https://orcid.org/0000-0002-7412-0297?lang=en)

**Address of corresponding and first author:**

Stanford University

Molecular Imaging Program at Stanford (MIPS), Department of Radiology

1701 Page Mill Rd, Palo Alto, CA 94304, USA

**General Information**

The Fmoc-(9-fluorenylmethoxycarbonyl-) and all other protected amino acid analogs were purchased from Thermo Fisher Scientific Inc. (Waltham, MA, USA) or VWR International (Radnor, PA, USA). The PS-Rink amide AM resin (100-200 mesh particle size, 0.6-0.7 mmol/g loading) was purchased from VWR International (Radnor, PA, USA). DOTA(*^t^*Bu)_3_ was purchased from Macrocyclics Inc. (Plano, TX, USA). Peptide syringes (20 mL with frit) were obtained from Thermo Fisher Scientific Inc. (Waltham, MA, USA).

All necessary solvents and other organic reagents were purchased either from Thermo Fisher Scientific Inc. (Waltham, MA, USA) or VWR International (Radnor, PA, USA). Solid-phase synthesis of the peptides was carried out by manual operation using a SCILOGEX SCI-RD-Pro LCD Digital Tube Rotator (Scilogex^®^, Rocky Hill, CT, USA). H_2_O was used after purification by a MilliporeSigma™ Synergy™ Ultrapure Water Purification System (Thermo Fisher Scientific Inc., Waltham, MA, USA).

Analytical and preparative reversed-phase high performance liquid chromatography (RP-HPLC) were performed using an Agilent 1260 Infinity II gradient system (Agilent Technologies, Santa Clara, CA, USA), equipped with a 1260 Quat Pump VL and a 1260 Infinity II Multiple Wavelength Detector (220 nm, 254 nm). Different gradients of MeCN (+0.1% TFA) in H_2_O (+0.1% TFA) were used as eluents for all RP-HPLC operations.

Preparative RP-HPLC purification was done using a Luna^®^ 5 µm C18(2) 100 Å LC column (250 × 21.2 mm, Phenomenex Inc., Torrance, CA, USA) at a constant flow rate of 10 mL/min.

Electrospray ionization-mass spectra for characterization of the substances were acquired on an expression^L^ compact mass spectrometer (Ithaca, NY, USA).

Lyophilization was accomplished using a Labconco™ FreeZone™ 6L Console Freeze Dryer (Labconco Corporation, Kansas City, MO, USA).

Solutions of purified compounds were prepared using Invitrogen^TM^ UltraPure^TM^ DNase/RNase-Free Distilled Water (Thermo Fisher Scientific Inc., Waltham, MA, USA).

For radio thin layer chromatography, a Dual Scan-RAM^TM^ Dual Scan-RAM radio-TLC and radio-HPLC System with Laura^TM^ Radiochromatography Data Collection and Analysis Software (LabLogic Systems Ltd., Broomhill, Sheffield, United Kingdom) was used.

***Cell Culture***

GRPR^+^ PC-3 cells (CRL-1435, ATCC, Manassas, VA, USA) were cultured in VWR^®^ Tissue Culture Flasks (Avantor Sciences, Radnor, PA, USA) using F-12K, 1X (Ham’s F-12K Nutrient Mixture, Kaighn’s Mod.) with L-glutamine (Corning, Sunnyvale, CA, USA) supplemented with Gibco^TM^ Fetal Bovine Serum, dialyzed, US origin (10%, v/v, Thermo Fisher Scientific Inc., Waltham, MA, USA) and Gibco^TM^ Anti-Anti (1%, v/v, Antibiotic-Antimycotic (100X), Thermo Fisher Scientific Inc., Waltham, MA, USA) as medium inside a 1300 Series Class II, Type A2 Biological Safety Cabinet (Thermo Fisher Scientific Inc., Waltham, MA, USA), and maintained in a Forma™ Steri-Cycle™ CO_2_ Incubator (Thermo Fisher Scientific Inc., Waltham, MA, USA) at 37°C in a humidified 5% CO_2_ atmosphere.

Gibco^TM^ TrypLE^TM^ Express Enzyme (1X), no phenol red (Thermo Fisher Scientific Inc., Waltham, MA, USA) was used to harvest cells. Cells were counted by a Invitrogen^TM^ Countess 3 FL automated cell counter (Thermo Fisher Scientific Inc., Waltham, MA, USA) using Gibco^TM^ Trypan Blue Stain (0.4%, v/v, in a 0.85% saline solution) solution (Thermo Fisher Scientific Inc., Waltham, MA, USA) on a Invitrogen^TM^ Countess^TM^ cell counting chamber slide (Thermo Fisher Scientific Inc., Waltham, MA, USA).

**Supplemental Data**

**SUPPLEMENTAL FIGURE 1.** Serum parameters of healthy Nu/J mice sacrificed at 45 d after injection of [^177^Lu]Lu-RM2, [^177^Lu]Lu-AMTG, [^161^Tb]Tb-RM2, and [^161^Tb]Tb-AMTG, as well as of the control group (no radioactivity received). Data expressed as mean ± SD.
